# Sex and Relationship Education for the Autonomy and Emotional Well-Being of Young People

**DOI:** 10.3389/fpsyg.2020.01280

**Published:** 2020-06-19

**Authors:** Rosa Marí-Ytarte, Roberto Moreno-López, Rut Barranco-Barroso

**Affiliations:** Department of Pedagogy, University of Castilla-La Mancha, Education and Society Research Group (GIES), Talavera de la Reina, Spain

**Keywords:** young people, autonomy, emotional well-being, relationships, sexuality, education

## Abstract

In the transition to adulthood, sexuality and emotional relationships constitute one of the most important dimensions for the achievement of personal autonomy and emotional well-being. Despite advances in sex education, sexuality, and relationships remain conflictive areas in the development of young people. Inequalities between men and women, gender identities and sexual violence, along with the beliefs and expectations surrounding these issues, persist as handicaps to having a fulfilling relationship and sex life. At this stage, emotional well-being is also consolidated by one’s perception of sexuality and relationships from models learned in childhood, in which gender stereotypes and sexuality based on relationships of domination and discrimination persist. Therefore, we examined how the sexual beliefs and practices reported by young people correlate with their level of personal autonomy and responsibility in terms of risky behaviors and toxic relationships. The study shows the extent to which sexual beliefs and habits are linked to decision-making, personal development and social problems derived from conflictive relationships, affecting young people’s overall well-being. A questionnaire was developed based on the theoretical constructs of comprehensive sexuality and equality education (United Nations Educational Scientific and Cultural Organization [UNESCO], 2010, 2018; World Health Organization [WHO], 2010) with the following dimensions: *sex education*, *sexual habits and practices*, *motivations*, *concepts*, and *beliefs about sexuality*. It was distributed in institutions of higher education (N579) in Spain, Portugal, Argentina, and Brazil, and the results revealed a discrepancy between the reported practices and behaviors and the beliefs and models of reference. Key issues included sexuality and relationships as an aspect of personal life that generates confusion and conflict, as well as the propagation of gender and sexist stereotypes that influence young people’s emotional well-being, particularly important aspect in those young people who are training as future education professional.

## Introduction

The World Health Organization (WHO) describes sexual health as a state of physical, emotional, mental, and social well-being in relation to sexuality; it is not merely the absence of disease, dysfunction or infirmity. Sexual health requires a positive and respectful approach to sexuality and sexual relationships, as well as the possibility of having pleasurable and safe sexual experiences, free of coercion, discrimination, and violence. For sexual health to be attained and maintained, the sexual rights of all persons must be respected, protected, and fulfilled (World Health Organization [WHO], 2002, p. 5).

Sexual health is therefore a permanent experience of physical, psychological and sociocultural well-being. Such is the case that we can observe subjects’ level of sexual health based on their responsible sexual behaviors. The latter include not only safe sexual relations, but also sexual behaviors in which autonomy, maturity, honesty, respect, consent, protection, and the pursuit of well-being come into play.

It is currently argued that sexuality is a basic human function involving physiological, emotional, and cognitive factors and connected to other personal and psychological aspects such as well-being, health and quality of life. In this study, we understand sexual satisfaction as the level of pleasure, well-being, and adjustment observed during a sexual interaction (Rodríguez, 2010).

Sexual satisfaction constitutes a human right and a key element of quality of life associated with a better state of physical and mental health (Whipple et al., 2003; Moore and Davidson, 2006; Levin, 2007; Scott et al., 2012). Connections between sexual satisfaction, physical health, psychological health, general well-being (Scott et al., 2012), and quality of life (Davidson et al., 2009) have been identified.

Also, the relationship between sexual satisfaction and psychological variables, including body image and self-esteem, has been widely studied. In this regard, higher self-esteem has been found to be associated with lower body dissatisfaction (Mellor et al., 2010; Salvador et al., 2010). When the relationship between sexual satisfaction and self-esteem has been explored, positive correlations have been found between these variables (Calado et al., 2004).

Unawareness of the emotional, mental, and psychological aspects of sexual health is highly problematic. If young people are uninformed of the importance of these aspects and of sexual pleasure and how that pleasure can be achieved and maintained, they may, as evidenced by the findings of Bakker and Vanwesenbeeck (2006), fail to seek help in the face of problems with sexual function. In the absence of a broader education on sexual health, young people are likely to perceive sexual problems as normal and run the risk of experiencing physical and psychological side effects.

The United Nations Educational Scientific and Cultural Organization [UNESCO], 2010 highlights the need to implement effective sexuality education, given that the cultural values and religious beliefs of all people (and particularly young people) have a major impact on the understanding of this issue and on relationships with other adults and their communities. Comprehensive sexuality education (CSE) responds to this demand, empowering young people to make informed decisions about relationships and sexuality while providing them with the necessary knowledge and skills to lead a satisfactory personal, social, and sex life.

In general, a sense of well-being is an indicator of quality of life that may be understood as the result of the perception of quality of life. In other words, well-being is an interpretation of quality of life based on subjective experience and environmental and personal filters (Langlois and Anderson, 2002). And psychological well-being emphasizes the attainment of values that make people feel alive and authentic, that allow them to grow as individuals, more than activities that give them pleasure or take away pain (Zubieta et al., 2012). Psychological well-being focuses on the study of personal growth, positive experiences, subjective well-being or happiness level and the optimal functioning of people, communities, and society (Duckworth et al., 2005; Snyder and Lopez, 2005).

The study of sexuality and its relationship with well-being has been a recurring research theme for decades (MunÞoz and Revenga, 2005; Borrego and Enriìquez, 2013). Also, in its 2010 report, the WHO recommends appropriate sex education for individuals’ level of development, based on gender equality, self-determination and acceptance of diversity and backed by scientific knowledge (Martiìnez-Aìlvarez et al., 2012). However, it was not until a few years ago that scientific and academic interest in issues such as gender experienced a considerable boom, due largely to the crusade undertaken by multiple social movements (Platero and Rosón, 2012).

Knowledge of and attitudes toward sexuality adopted as an individual matures are largely derived from what is transmitted by the different social-educational contexts to which the subject is exposed during his or her socialization process. On the other hand, the vast majority of studies have focused almost exclusively on the behavioral component of sexuality, such as types of sexual behavior, contraceptive use, risks, number of partners, etc. (Santín et al., 2003; Alencar et al., 2008; Altmann, 2009; Andrade et al., 2009; García-Vega et al., 2010, 2012; Kornblit and Ezequiel, 2015; Martín et al., 2018; Nascimento et al., 2018).

Scientific literature shows that young people adopt liberal, erotophilic attitudes toward sexuality, and especially toward what is more socially visible and acceptable, such as heterosexuality or coitus (García-Vega et al., 2017). However, their attitudes are less positive toward other issues not linked to the dominant norm, such as some sexual practices or the expression of sexual and gender diversity (Claramunt and Huertas, 1999). In addition, numerous stereotypes and myths associated with sexuality are perpetuated due to a lack of education, the view of sexuality as taboo and the predominance of normative values associated with conservative and religious morality (López, 2015).

In line with the above, it appears that overall, young people continue to assign stereotypical roles to men and women in relation to their experience of sexuality. In this regard, Sierra et al. (2007) studied the attitudes of 400 Spanish university students toward sexuality and concluded that; in general, sexist attitudes persist in which the possibility of gender equality is not considered.

Several studies have found gender differences in terms of both behaviors and feelings around sexuality, where women seem more focused on emotional issues and men on sexual behaviors (Friedrich et al., 2000; Faílde et al., 2008; López et al., 2011; Larrañaga et al., 2012). Men begin having sexual relations sooner and have more sex partners and more casual encounters (Faílde et al., 2008; Petersen and Hyde, 2010). In addition, having sexual relations with different partners continues to be seen more positively by men than by women (Crawford and Popp, 2003).

In this sense, many differences are rooted in the different socialization processes for men and women, which demonstrate the importance of considering sociocultural factors when analyzing such differences (Li et al., 2017).

In addition, gender and the experience thereof, or of non-normative sexuality, appears as one of the main focuses of discrimination, especially in educational contexts (Generelo, 2016). In this sense, discrimination based on sex or gender is maintained by its invisibility or by maintaining active discriminatory behaviors (European Union Agency for Fundamental Rights, 2014).

At this time, sex education generally pivots on the coexistence of the moral-conservative model and the risk model (Carrera-Fernández et al., 2012). Therefore, it is essential that a high-quality relationship and sex education that contemplates sexuality as an inherent aspect of the human being (Fallas-Vargas et al., 2012; Rodríguez-Carrión and Traverso-Blanco, 2012) be integrated into the core educational process. Sex education is an essential support for the achievement of well-being and quality of life, as well as a right whose exercise allows informed decisions to be made (World Health Organization [WHO], 2010).

The World Health Organization [WHO], 2018 recommends implementing CSE programs that allow subjects to enjoy health, well-being and dignity, becoming aware of how the decisions they make affect not only their own well-being, but also that of other people. Meanwhile, the United Nations Educational Scientific and Cultural Organization [UNESCO] (2018) states that CSE programs are central to preparing young people for a safe, productive and full life. Marí et al. (2019) point out the importance of acquiring through these programs skills and competencies that allow subjects to discover and develop their own autonomy and well-being in relation to others, where sexuality, self-knowledge and personal relationships are points of reference to express themselves as well-rounded human beings. By promoting positive relationships between the subject and his or her surroundings, they promote positive, healthy changes, leaving behind the sex education models focused on the presence or absence of disease (Viejo et al., 2018).

## Methodology

Our initial hypothesis is that the sex education young people receive and the beliefs and sexual behaviors derived from it have an impact on their emotional well-being as individuals and in their social relationships. We start with the premise that, as indicated by WHO and UNESCO, sexuality is a part of and necessary for emotional well-being in youth, influencing aspects related to health, social bonds, and personal autonomy. In addition, we agree that sexuality develops and is in turn influenced by the perceptions that young people have of it, based on educational models on topics such as *ideology*, *gender equality*, *emotional bonds*, and *social relationships*. Therefore, emotional well-being at this stage of life is presumably subject to a double need: *the construction of personal identity* (with sexuality as an important dimension of it) and *social acceptance*, especially by one’s peer group. In the study, we explore the extent to which young people’s perceptions, practices, and beliefs about their sexuality converge with those recognized by the WHO and UNESCO as positive for sexual fulfillment and emotional well-being at this stage of life.

Following the WHO’s reports we share evidence that sexuality remains a social taboo that, in most societies, prevents educational institutions from providing a comprehensive education from early childhood that would enable one to approach one’s first sexual relations with confidence, autonomy, and security. It is also observed that gender inequalities and the greater social vulnerability of girls persist as a problematic or risk factor in sexual relations. Taboos, preconceived ideas about what sex is and an incomplete sex education, or one limited to aspects concerning reproduction or health protection, have been identified. According to these reports, a heteronormative discourse persists that focuses mainly on genitality while excluding a positive, diverse view of sexuality that allows young adults to grow from their own sexual experiences confidently and autonomously.

The WHO and UNESCO indicate that sexuality affects young people’s emotional well-being because relationships can become problematic if sexual experiences generate personal conflict, insecurity or submission to social models that contradict a young person’s own identity, due to:

1. A lack of information and of a thorough, high-quality education.
2. Mental maps and distorted beliefs about sexuality.
3. Inappropriate or risky behaviors due to a lack of education, communication, and autonomy.
4. The (distorted) sexualization of society, which implies its genitalization, its masculinization (women as objects), the dichotomy between sexual and emotional relationships and the linkage of the latter to the stable partnership.

### Study Population and Sample

1. The sample focuses on the university population of two countries in Europe (Spain 26.3%; Portugal 20.5%) and two countries in Latin America (Brazil 35.3%; Argentina 17.9%), with a total of 579 respondents among university students in 2017. The following parameters were established for the representative study sample: sampling error of ±2.7 sigmas, confidence level of 95%. The questionnaire was completed by a larger number of women (male representation was 4.3%, an aspect that limited the interpretation of the results from a gender perspective and the explanatory nature of the sex variable (therefore the different weight of the sample does not allow for a gender analysis). The selection of this sample regarding its autonomy and emotional well-being linked to sexuality is defined by the great importance of this group, as future trainers and workers with children and teenagers since, whether or not explicitly, they will contribute through their practice to the sex education of the next generations. From a gender perspective, encompasses much more than biological sexual division. We have tried to show this in the dimensions studied, especially those relating to affective beliefs, behaviors, and relationships, and how gender identity and its expression is strongly linked to emotional and social well-being at this stage of life. Regarding age, 54.1% of the participants were in the under 25 age group and 45.9% were over the age of 25. The criteria used to define the study sample were: (a) the minimum range: over 18 years old, since they were university students. (b) Regarding the maximum strip: up to 30 years. Being the most unifying criterion in most countries, which in the case of Argentina (National Youth Directorate) and Brazil (National Secretary of Youth) would be 29 years and in the case of Spain and Portugal (EU) would be 30 years, as pointed out in the European Union Strategy for Youth 2019–2027. According to the IEO, an Organization for Ibero-American States, which also includes Spain and Portugal, the United Nations defined youth in 1985 between the ages of 15 and 24. However, according to the 2008 report, which came into force, the Ibero-American Convention on Youth Rights (CIDJ) came into force, it is noted that in Europe the age is up to 29–30 years (due to the prolongation of schooling and the delay of the age of formation of own families).

### Instrument, Categories and Analysis Procedure

The study was carried out through an *ad hoc* questionnaire based on the categories defined by the WHO and UNESCO regarding emotional well-being linked to sexuality in young adulthood.

• Education received.
• Sexual practices and habits.
• Beliefs and expectations regarding sexuality.
• Emotional relationships.

WHO’s categories define emotional well-being and mental health around sexuality within socio-cultural framework that influences each of the above dimensions in young people’s personal development, such as:

• The social and cultural norms of the environment (beliefs, religion, and politics).
• The sociopolitical context (human rights and legislation).
• Gender or sociocultural inequalities (education level and gender).
• Sexual function and psychosexual orientation.
• Policies to prevent gender-based violence and aid its victims and to prevent and control the spread of HIV and other sexually transmitted diseases.

For its part, the UNESCO defines sex education as a necessary resource backed by scientific evidence and aimed at promoting young people’s health and well-being. In this regard, emotional well-being arising from sex education would be founded on:

• Comprehensive sex education backed by proven knowledge.
• Development of a sex-positive attitude.
• Safe sex practices and access to contraceptive methods.
• Incorporation of the gender perspective.

Based on these premises, we configured the dimensions of analysis for the data collection instrument and the variables that represent each dimension studied. This allowed us to examine young people’s perceptions of each one in relation to the categories that define emotional well-being (WHO), as illustrated by the following list:

Dimension 1. Education sources: level and experience of sex education received. Associated variables:

1. *Educasex*: information and instruction on sexuality received in formal and informal educational contexts.
2. *Personaldev*: impact of education received on personal development.
3. *Informalcompare*: comparison of categories 1 and 2.

Dimension 2. Habits: sexual behaviors and practices. Associated variables:

4. *Firstsex*: first sexual relations.
5. *Numpartners*: number of partners to date.
6. *Freqsex*: frequency of sexual practices.
7. *Orgasm*: relationship between sexuality and pleasure.
8. *Contraceptives*: safe sex and/or risky habits and practices.
9. *Masturbation*: practices and perception of autoerotism.
10. *Fidelity*: association of sexuality with stable partner relationships.
11. *Samesex*: perception and assessment of sexual relations with same-sex partners.

Dimension 3. Motivations: expectations of sexual relations. Associated variables:

12. *Attractiveness*. Qualities of the other person.
13. *Personalpleasure*. Seeking one’s own satisfaction.
14. *Otherperspleasure*. Giving pleasure.
15. *Bothpleasure*. Giving and receiving pleasure.
16. *Emoinvolve*. Emotional involvement and intimate bonds.
17. *Reproduction*. Pregnancy and offspring.

Dimension 4. Sexual and gender identity concept: perception of sexual diversity. Associated variables:

18. *Sexualiden*. Sexual identity.
19. *Gay*. Homosexuality.
20. *Lesbian*. Lesbianism.
21. *Transex*. Transsexuality.
22. *Ofgender*. Gender identity.
23. *Bisexual*. Sexual relations with members of either sex.
24. *Sexism*. Prejudices based on gender.
25. *Homophobia*. Prejudices toward sexuality with same-sex individuals.
26. *Transphobia*. Prejudices toward transsexuality.

Dimension. 5. Values regarding sexuality. Beliefs and values regarding sexuality.

Associated variables:

27. *Varioussex*. Having sexual relations with different partners at the same time.
28. *Lesscommit*. Dissociation of sexuality and emotional or committed relationships.
29. *Sexproblem*. Promiscuity as a source of problems in relationships.
30. *Immoralmasturb*. Masturbation as a reprehensible practice.
31. *Premarital*. Assessment of sexual relations before marriage or stable partnership.
32. *Withpeople*. Experience and assessment of sexual relations with same-sex partners.
33. *Prostitut*. Perception and assessment of prostitution.
34. *Eroticmat*. Perception and assessment of the use of erotic toys.
35. *Sexlove*. Assessment of the link between sexuality and emotional bonds.
36. *Otheroptions*. Respect for sexual diversity.
37. *Sexpleasure*. Assessment of pleasure in sexual relations.
38. *Menmore*. Perception of the importance of sexuality linked to biological sex.

First, a descriptive analysis was conducted to calculate the contrast of means in order to describe the behavior of each dimension’s variables in relation to the differentiating variables based on sex, age group, and continent. After this descriptive analysis, we performed the correlation analysis (Pearson’s *r* coefficient), where we determined a possible relationship between the different variables designed for each of the five components of well-being according to the WHO and UNESCO criteria. The probability value established for this study was *p* < 0.05. Finally, we performed a factor analysis of rotated components and principal components and KMO test in order to determine the main components for the development of discourses within the predictive rotation of the relationship between variables. Regarding the analysis procedure, descriptive statistics were prepared with the IBM SPSS statistics 22 program.

## Results

### Contrast of Means Results

After applying the contrast of means across all dimensions and their variables, and with age and continent serving as differentiating variables of the mean, the results were statistically significant to our study. The results exhibit significance in all variables and all cases with regards to differences by age group and by continent.

#### Dimension 1. Education Sources: Level and Experience of Sex Education Received

In particular, all categories associated with the Education dimension have a perfect result and, as illustrated in Table 1, the young women especially value the formal education received above learning in informal contexts, particularly in Argentina and Brazil. Likewise, individuals under the age of 25 respond positively to this education. Individuals over the age of 25 (1.78) and Europeans (1.78) attribute greater importance to academic education than individuals under the age of 25 and Latin Americans (1.39). In contrast, individuals over the age of 25 (1.64) and young Latin American women (1.77) assign greater importance to *sex education contribution to personal development*. Finally, in terms of formal versus informal sex education, by age group, to people over the age of 25 (1.76) than to younger respondents (1.63); and by continent, to Latin Americans (1.83) than to Europeans (1.62).

**TABLE 1:** Contrast of means – sex education sources.

	**Age group**			**Continent**		
		**Mean**	**Significance**		**Mean**	**Significance**
Academic sex education	<25	1.78	0.000	Eu	1.78	0.000
	>25	1.45		Am	1.39	
Contribution of sex education to personal development	<25	1.50	0.008	Eu	1.47	0.000
	>25	1.64		Am	1.77	
Formal sex education versus informal	<25	1.63	0.004	Eu	1.62	0.000
	>25	1.76		Am	1.83	

*Source: authors (1 = low well-being/2 = high well-being).*

#### Dimension 2. Habits: Sexual Behaviors and Practices

In terms of the sexual habits reported by the young people surveyed, statistical significance was found based on age group, the statistically significant variables were 5, 6, 7, and 8: *number of partners* (significance 0.001), *frequency of sexual relations* (significance 0.023), *orgasm* (significance 0.000), and *contraception* (significance 0.003). In addition, looking at differences by continent, the results have greater statistical significance in variables 5 and 6: significance 0.036 and 0.029, respectively. In other words, in all categories that clearly link sexuality to pleasure-seeking and personal satisfaction in all groups and countries.

#### Dimension 3. Motivations: Expectations of Sexual Relations

We found perfect significance levels in relation to age groups, we find important data in relation to variables 15 and 17: *pleasure of both* (significance 0.036) and *emotional involvement* (significance 0.032). Regarding continent of origin, we observe statistical significance in variables 12 and 15: *partner attractiveness* (significance 0.025) and *pleasure of both* (significance 0.005). In terms of personal motivation to have sexual relations, pleasure from the relations and the possibility of reproduction, are more important in this dimension.

#### Dimension 4. Sexual and Gender Identity Concept: Perception of Sexual Diversity

In the contrast of means, we find relevant results with perfect significance levels when differentiating by continent in variables 19 and 20: *gay concept* (significance 0.000) and *lesbian concept* (significance 0.000). Also noteworthy is the high statistical significance of variables 23 and 24: *bisexuality* (significance 0.037) and *sexism* (significance 0.002). If we differentiate by age groups, the significant variables are 19 and 23: *gay* (significance 0.005) and *bisexuality* (significance 0.045). In this regard, the results show a largely heteronormative, traditional perception of sexuality. Sexual diversity is accepted but barely represented as a value in one’s own sexual experience.

#### Dimension 5. Values Regarding Sexuality. Beliefs and Values Regarding Sexuality

The contrast of means yields relevant results when differentiating by continent in variables 28, 29, and 37: *better sexuality with less commitment* (significance 0.000), *youth promiscuity as problematic* (significance 0.001), and *sex linked to pleasure* (significance 0.008), which is also statistically significant with respect to age (significance 0.000). Regarding the variable 33 relating to *prostitution*, this is little accepted by the young women surveyed and that they give less importance to sex (variable 38). Beliefs and values regarding sexuality thus show, in all cases, that sexuality is perceived in terms of satisfaction and pleasure, and at the same time, that these variables are associated with the perception that women have about men.

### Correlation Results

We applied Pearson’s correlation analysis to determine the strength of the relationship between the described variables and the study dimensions. First, the results yielded a strong positive correlation between formal sex education versus informal and the contribution of sex education to personal development (*r* = 0.417; significance 0.000) (Table 2).

**TABLE 2:** Correlations for the education dimension.

		**Religion**	**Academic sex education**	**Formal sex education versus informal**
Contribution of sex education to personal development	Pearson correlation	0.105	−0.170	0.417
	Significance (one-tailed)	0.043	0.000	0.000
	*N*	270	545	545

*Source: authors.*

When the correlation between the variables comprising dimension 2 (*sexual habits*) is calculated, as illustrated in Table 3, the data has strong statistical significance for the relationship between *no. of partners* and *religion* (*r* = 0.0497; significance 0.000) and to a lesser extent, *age of first sexual relations* (*r* = 0.0266; significance 0.000), *frequency of sexual relations* (*r* = 0.235; significance 0.000), and *Orgasm* (*r* = 0.0224; significance 0.000). In the case of the *orgasm* variable, the only statistical significance concerns *frequency of sexual relations* (*r* = 0.239; significance 0.000) and to a lesser extent, *age of first sexual relations* (*r* = 0.161; significance 0.000). The rest of the variables do not show important correlations in this case.

**TABLE 3:** Correlations for the habits dimension.

		**Religion**	**Age first sex *R***	**Frequency sex *R***	**Orgasm in sex *R***
No. of partners	Pearson correlation	0.497	0.266	0.235	0.224
	Significance (one-tailed)	0.000	0.000	0.000	0.000
	*N*	289	579	579	579
Orgasm in sex *R*	Pearson correlation	−0.066	0.161	0.293	
	Significance (one-tailed)	0.133	0.000	0.000	
	*N*	289	579	579	

*Source: authors.*

Table 4 contains the correlation results for dimension 3 (*motivations*). The greatest statistical significance is observed between *partner’s pleasure* and *personal pleasure* (*r* = 0.404; significance 0.000). Also, a negative correlation appears in the case of *partner’s pleasure* and *reproduction* (*r* = −0.200; significance 0.000). The rest of the variables do not yield statistically significant results for this dimension.

**TABLE 4:** Correlations for the motivations dimension.

		**Personal pleasure**	**Reproduction**
Partner’s attractiveness	Pearson correlation	−0.146	0.094
	Significance (unilateral)	0.000	0.012
	*N*	579	579
Partner’s pleasure	Pearson correlation	0.404	−0.200
	Significance (unilateral)	0.000	0.000
	*N*	579	579

*Source: authors.*

In dimension 4 (*sexual and gender identity concepts*), we find a strong correlation between *lesbian concept* and *gay concept* (*r* = 0.781; significance 0.000), and to a lesser extent, between the *sexism concept* (*r* = 0.166; significance 0.042) and the *lesbian concept* variables. It is important to highlight the inverse correlation with the *gender identity concept* (*r* = −0.163; significance 0.044). When we focus on religion, the data is statistically significant for the *sexual identity concept* and the *gay concept* and to a lesser extent for the *transphobia concept* (*r* = 0.142; significance 0.155), as we can see in Table 5.

**TABLE 5:** Correlations for the sexual and gender identity concepts.

		**Sexual identity concept**	**Gay concept**	**Gender identity concept**	**Sexism concept**	**Transphobia concept**
Religion	Pearson correlation	0.251	0.230	0.055	−0.017	0.142
	Significance (one-tailed)	0.035	0.049	0.347	0.452	0.155
	*N*	53	53	53	53	53
Lesbian concept	Pearson correlation	−0.036	0.781	−0.163	0.166	0.047
	Significance (one-tailed)	0.354	0.000	0.044	0.042	0.314
	*N*	110	110	110	110	110

*Source: authors.*

Finally, in Table 6, the analysis of correlations for dimension 5 (*beliefs and values regarding sexuality*), we find a statistically significant relationship between *permissiveness toward occasional promiscuity* and *promiscuity as problematic* (*r* = 0.230; significance 0.000) and a negative correlation between the former and *permissiveness toward prostitution* (*r* = −0.219; significance 0.000). There is also a positive correlation between *premarital sex* and the *use of erotic materials* (*r* = 0.212; significance 0.000). There is a negative correlation between *permissiveness toward prostitution* and *premarital sex* (*r* = −0.181; significance 0.000), *use of erotic materials* (*r* = 0.207; significance 0.000), and *sex* = *love* (*r* = −0.210; significance 0.000). *Use of erotic materials* is directly related to *premarital sex* (*r* = 0.212; significance 0.000) and *sex associated with love* (*r* = 148; significance 0.000) and shows a negative correlation with *promiscuity as problematic* (*r* = −0.106; significance 0.005) and with *permissiveness toward prostitution* (*r* = −0.207; significance 0.000). The *sex* = *love* variable shows a strong correlation with *promiscuity as problematic* (*r* = 0.222; significance 0.000), *premarital sex* (*r* = 0.153; significance 0.000), and *use of erotic materials* (*r* = 0.148; significance 0.000) and a negative correlation with *permissiveness toward prostitution* (*r* = −0.210; significance 0.000).

**TABLE 6:** Correlations for the beliefs and values about sexuality dimension.

		**Youth promiscuity as problematic**	**Premarital sex**	**Permissiveness prostitution**	**Use of erotic materials**	**Sex = love**
Permissiveness toward occasional promiscuity	Pearson correlation	0.230	0.171	−0.291	0.080	0.245
	Significance (one-tailed)	0.000	0.000	0.000	0.027	0.000
Premarital sex	Pearson correlation	−0.001		−0.181	0.212	0.153
	Significance (one-tailed)	0.486		0.000	0.000	0.000
Permissiveness prostitution	Pearson correlation	−0.008	−0.181		−0.207	−0.210
	Significance (one-tailed)	0.423	0.000		0.000	0.000
Use of erotic materials	Pearson correlation	−0.106	0.212	−0.207		0.148
	Significance (one-tailed)	0.005	0.000	0.000		0.000
Sex = love	Pearson correlation	0.222	0.153	−0.210	0.148	
	Significance (one-tailed)	0.000	0.000	0.000	0.000	

*Source: authors.*

### Factor Analysis Results

After applying the factor analysis to each dimension to establish the possible discourses that appear in the predictive rotation of the relationship between each dimension’s variables (concerning potential differences based on age, country, and continent), statistically significant data was obtained.

As observed in Table 7, when we analyze the results relating to the first dimension, *education sources*, the data in the case of Europe and Spain, surpasses 75% of the variance explained. Spain follows that same trend being the country that makes the most difference in this issue.

**TABLE 7:** Factor analysis education dimension.

**Variable**	**% of variance explained**
Total matrix	0.489
≤25	0.476
>25	0.492
Spain	0.781
Portugal	0.512
Brazil	0.439
Argentina	0.525
Europe	0.796
Ibero-America	0.564

*Source: authors.*

If we focus on the second dimension, *sexual habits*, only Portugal and Brazil (and the two South American countries together) surpass 65% of the rotation (Table 8).

**TABLE 8:** Factor analysis habits dimension.

**Variable**	**% of variance explained**
Total matrix	0.525
≤25	0.406
>25	0.527
Spain	0.421
Portugal	0.674
Brazil	0.696
Argentina	0.655
Europe	0.415
Ibero-America	0.651

*Source: authors.*

However, in the case of dimension 3, *motivations*, Portugal and Argentina are the ones that approach 75% of the variance explained (Table 9).

**TABLE 9:** Factor analysis motivations dimension.

**Variable**	**% of variance explained**
Total matrix	0.466
≤25	0.645
>25	0.641
Spain	0.472
Portugal	0.736
Brazil	0.573
Argentina	0.750
Europe	0.472
Ibero-America	0.647

*Source: authors.*

Regarding dimension 4, *gender and sexuality concept*, it should be noted that all elements approach 60% (Table 10).

**TABLE 10:** Factor analysis gender and sexuality concept.

**Variable**	**% of variance explained**
Total matrix	0.621
≤25	0.629
>25	0.633
Spain	0.521
Portugal	0.658
Brazil	0.632
Argentina	0.697
Europe	0.627
Ibero-America	0.633

*Source: authors.*

Finally, when we look at *values and beliefs*, the case of Argentina stands out at more than 70% of the variance explained (Table 11).

**TABLE 11:** Factor analysis values – beliefs dimension.

**Variable**	**% of variance explained**
Total matrix	0.466
≤25	0.474
>25	0.669
Spain	0.479
Portugal	0.658
Brazil	0.531
Argentina	0.732
Europe	0.471
Ibero-America	0.525

*Source: authors.*

### Results of Factor Analysis by Components

#### Dimension 1. Education Sources

The component that appears in the total matrix introduces variables 1, 2, and 3, *academic sex education*, *contribution of sex education to personal development*, and *assessment of formal versus informal learning* as significant, with a % of variance explained of 0.489. In the University students, variables 2 and 3 stand out as the first component, the item’s correlation weight shows a negative factor, the % of variance is explained by 0.0490. By ages and in all cases, the first component includes the three variables concerning the importance of education received. This variable is also the first component in three of the four countries studied, and it is not valued in Spain.

#### Dimension 2. Habits: Sexual Behaviors and Practices

Regarding sexual habits, the total matrix shows the three components as clearly differentiated. The first with respect to variables 5, 7, and 6: *frequency of sexual relations*, *orgasm*, and *number of partners*. The second concerns variables 10, 11, and 9, *fidelity*, *sexual relations with same-sex partners*, and *masturbation*; and the third relates to variables 8 and 4, *contraceptive use* and *age of first sexual relations*. On the other hand, for the young women surveyed, significant differences can be seen in the importance attached to *orgasm* and the *frequency of sexual relations*, leaving component 9, which concerns masturbation, to the second component, as less significant. *Contraceptive use* is the third component.

In this dimension, the differences based on age should be noted: while respondents under the age of 25 emphasize variables 5, 7, 6, and 4 (*number of partners*, *orgasm*, *frequency of sexual relations*, and *age of first sexual relations*) first, those over the age of 25 highlight variables 11, 9, and 10 (*relations with same-sex partners*, *masturbation*, and *relations with third parties*), and 11 and 9 correspond to the second component for those under that age. The second component is associated with *first sexual relations*, *number of partners*, and *contraceptive use*, and the third with *orgasm* and *frequency of sexual relations*.

When comparing countries, the most important components in Portugal and Spain were variables 5, 7, 4, and 6 (*number of partners*, *orgasm*, *first sexual relations*, and *frequency of relations*), while in Brazil and Argentina, they were variables 10 and 1 (*relations with third parties* and *sexual relations with same-sex partners*). Also noteworthy are the differences among countries regarding the perspective on contraceptive use, which is the second component in Spain and Portugal and the third in Brazil and Argentina.

#### Dimension 3. Motivations: Expectations of Sexual Relations

Regarding habits, the total matrix exhibits a single component with variables 14, 13, 17, and 12 (*other person’s pleasure*, *personal pleasure*, *reproduction*, and *partner’s attractiveness*). By age groups, respondents under the age of 25 considered the other person’s pleasure to be more relevant, followed by personal pleasure and reproduction in the third place. For those over the age of 25, aside from the other person’s pleasure, personal pleasure stood out compared to the other group. In all countries, the other person’s and personal pleasure were emphasized, followed by reproduction and attractiveness as first components, with slight differences in the order of importance. In Brazil and Argentina, more importance was assigned to the partner’s attractiveness. In all countries, emotional involvement in sexual relations appeared as the second component.

#### Dimension 4. Sexual and Gender Identity Concept: Perception of Sexual Diversity

In the total matrix, we find four components: the first with *gay and lesbian*, the second with *transphobia and bisexuality*, the third with *homophobia and sexism*, and the fourth with the *sexual identity*, *transsexuality*, and *gender inequality* concepts. For the first component, the people surveyed emphasize the *lesbian* and *gay* concepts. In the second component, *sexism* and *homophobia* stand. The third component corresponds to *sexual identity* and *bisexuality*. For the fourth component, overlap with *gender inequality*, and the latter add *transphobia*.

Looking at ages, it is important to note that younger respondents also emphasize variables 20 and 19, *lesbian* and *gay*, as the first component, although they diverge in the second component, as variables 26, 23, and 22 (*transphobia concept*, *bisexuality*, and *gender inequality*) are relevant to those under the age of 25, versus variables 25, 24, and 18 (*homophobia*, *sexism*, and *sexual identity*) for older respondents. Likewise, in the third and fourth components, differences based on age are observed. In the third, the relevance of sexism and homophobia stand out for those under the age of 25, versus sexual identity and transsexuality for the older group. In the fourth component, sexual identity and transsexuality are highlighted for those under 25, versus gender identities and transphobia for older respondents.

All countries also coincide with *lesbian and gay* concepts in the first component. For the second component, Spain and Portugal coincide with *homophobia*, *sexism*, and *gender inequality*, although Spain adds the *transphobia* concept. Brazil exhibits *gender inequality* and *transsexuality* versus Argentina with *homophobia*, *transphobia*, and *transsexuality*.

#### Dimension 5. Values Regarding Sexuality. Beliefs and Values Regarding Sexuality

The values of the total matrix exhibit two clear components. The first one corresponds to variables 34, 33, 31, and 37 (*use of erotic materials*, *permissiveness toward prostitution*, *premarital sex*, *and sex for pleasure and not reproduction*). The second component relates to variables 29, 36, 38, and 35: *promiscuity as problematic*, *respect for sexual diversity*, *greater male sexuality*, and *sexuality associated with love*; along with the assessment of premarital sex and sex associated with pleasure. As for the second component, the students indicate: *permissiveness toward prostitution*, *use of erotic materials*, and *promiscuity as problematic, casual relations*. And the viewpoint that *men have greater sexuality* define this area, with *respect for sexual diversity* corresponding to the third component. In fourth place, a fourth component appears concerning pleasure and love with variables 28, 37, and 35.

In the two age groups, those under the age of 25 exhibited in the first component more permissiveness toward occasional promiscuity and a stronger association of sexuality with pleasure and love, but not with reproduction. However, in this regard, the older group emphasized promiscuity as problematic and greater sexual activity in men. Secondly, the variables relating to the use of erotic toys, sex with third parties, and premarital relations (variables 34, 27, and 31) appeared as significant in both groups.

In terms of responses by country, the first component is the acceptance of casual relations with other people (variable 27) and a higher appreciation of sex associated with love (variable 35) but not reproduction. Prostitution is not viewed negatively in any country, and permissiveness toward its practice is observed in all of them. There is a positive assessment of sexual diversity (variable 36), although it appears in the third or fourth component in all of them. In both Spain and Portugal, the first component corresponds to permissiveness toward casual relations and the link between sex and love, which is the third component in Brazil and Argentina, which value the use of erotic materials (variable 34 in Brazil), the masculinization of sexuality (variable 38), and the problem of promiscuity (variable 29) as the first component.

## Discussion: Perception and Experience of Sexuality Among Young People

*The following is a discussion of the results in each dimension of our study as they relate to the emotional well-being of young people and the categories based on the WHO’s recommendations (2010, 2018). The latter are linked to the sex education that the United Nations Educational Scientific and Cultural Organization [UNESCO] (2009) studies and other recent research have found to be important to young people’s development in terms of sexuality*.

### Dimension 1. Education Sources: Level and Experience of Sex Education Received

Our study shows the importance and assessment of the sex education received by the young people we surveyed in four countries. In all cases, this instruction was provided mainly in secondary school and considered significant for personal development. It is relevant that when asked about the value of academic education versus information/learning obtained in informal contexts, the respondents clearly placed more value on the latter from the perspective of well-being and development, and this was especially the case of those under the age of 25. As the United Nations Educational Scientific and Cultural Organization [UNESCO] (2009) indicates, a comprehensive education based on scientific evidence is necessary from early childhood to encourage greater autonomy and safety during young adulthood. In this regard, education defines a higher degree of emotional well-being by providing more knowledge and a *positive attitude toward sexuality*.

### Dimension 2. Habits: Sexual Behaviors and Practices

In general, concerning sexual behaviors and habits, the results reveal a clear link between sexuality and the pursuit of pleasure and personal satisfaction in terms of the frequency of sexual relations and the number of partners; these aspects are more relevant than those linked to reproduction or stable relationships. Orgasm and autoerotism were key references in the minds of young people in this dimension. Masturbation appears as a habitual and recognized practice, although, as we will see in dimension 5 concerning values and beliefs about sexuality, it is viewed less positively. It should also be noted that prevention or contraceptive use was not a central or relevant theme for sexual habits, although it was more marked for those under the age of 25. Although one of the factors of emotional well-being around sexuality, according to the WHO, concerns prevention, including both safe sex and contraceptive use, the study reveals that risky habits persist in the youth population surveyed. This is linked to sexuality in which the frequency of sexual intercourse and the number of partners prevail over safety in the pursuit of satisfaction. In this regard, when observed in detail, there are some clear differences in sexual habits among the countries in our study, reflected in both the component and correlation tests: while in Portugal and Spain, frequency and number of partners are key aspects of sexual behavior, in Brazil and Argentina, relations with third parties and homosexual relations stand out first, although religion and, to a lesser extent, the age of first sexual relations have a significant correlation with perceived sexual behaviors. In this regard, the study showed that most young people have their first sexual experiences between the ages of 15 and 18. Following the guidelines of the United Nations Educational Scientific and Cultural Organization [UNESCO] (2018); Fonner et al. (2014), educational programs designed to provide more information combined with instruction that delays the age of first sexual relations result in safer practices. It should be noted that early sexual relations carry a higher probability of risky behaviors and unsafe practices and reduce emotional well-being for the young person in the long term.

### Dimension 3. Motivations: Expectations of Sexual Relations

According to the results of our study, in terms of motivations, the most emphasized aspects concern to one’s own satisfaction and pleasure and that of one’s partner, together with emotional bonds, and to a lesser degree, reproduction, which correlates negatively with sexual satisfaction. In this sense, emotional factors were more important to women, a fact that coincides with the results of several recent studies (Friedrich et al., 2000; Larrañaga et al., 2012; López et al., 2011). The young students showed a stronger inclination toward emotional and relationship and valued that men are more directly oriented toward excitement and pleasure (Faílde et al., 2008). However, reproduction was an inverse factor to pleasure, exhibiting a clear separation of these two aspects for all the young people surveyed.

In different countries, emotional involvement appears as a second component to shared pleasure with one’s partner and the importance of giving pleasure in the case of women. Own orgasm was equally important for all age groups, yet more significant in those under the age of 25. It should be noted that the attractiveness of one’s partner did stand out more as a motivation for sexual relations in countries such as Brazil and Argentina. Motivations toward sexuality reveal the extent to which young people perceive sexual function as an important dimension of their relationships. In this regard, one of the categories established by the WHO for young people’s emotional well-being around sexuality links motivations to the expectations learned in the social and community context (*WHO recommendations on adolescent sexual health and reproductive health and rights*, 2018 and the importance of the environment in encouraging young people’s habits and behaviors. Therefore, beyond the heterogeneity exhibited by the different countries in our study, CSE takes on great importance in this area, as it defines aspects concerning gender differences and the importance of emotional bonds and reproduction. This makes an impact on young people’s emotional development and safety, especially at a stage of life in which these aspects seem less important to them.

### Dimension 4. Sexual and Gender Identity Concept: How Sexual Diversity Is Perceived

The results of the questionnaire indicate that the perception of sexuality and emotional-sexual diversity and gender identity is quite varied, containing some heteronormative biases. Although sexual diversity is accepted, it is barely represented as a value in one’s own sexual experience. Young people adopt liberal attitudes toward sexuality and especially toward what is more socially visible and acceptable (García-Vega et al., 2017). The discourse of young university students about sexual diversity is becoming more variable and dynamic, perhaps because they are beginning to accept and integrate behaviors that fall outside of the norm dictated by the patriarchy or dominant hegemony. However, we can still see that transsexuality remains hidden and barely accepted, potentially, as López (2005) points out, due to the predominance of normative values associated with conservative morals.

Also noteworthy is the prevalence of low-intensity sexism, given that the variables related to gender inequalities did not have a prevalent place among women in any of the countries analyzed, appearing only as a secondary component in the tests performed. The fact that the study also reveals a certain degree of permissiveness toward prostitution indicates that young women are more vulnerable than men in their relationships, which can impact their health, safety, and personal well-being. This is a sign of the emergence of new scenarios of vulnerability for young people in increasingly globalized societies, with gender violence and sexual exploitation as some of their most worrisome manifestations (Melendro et al., 2018). The World Health Organization [WHO] (2018) points out in this regard that the permanence of conditions of social inequality increases violence toward women, especially at this stage of life. Therefore, all its recommendations include the gender perspective in sex education starting in childhood.

### Dimension 5. Values Regarding Sexuality. Beliefs and Values Regarding Sexuality

Finally, in terms of young people’s beliefs about sexuality, the results of this study indicate that sexuality is far more associated with pleasure and love than with reproduction. In modern society, according to Beck and Beck-Gernsheim (2001), there is no competent external moral authority on matters of love. Sexuality occupies a space of its own within the process of defining one’s identity and as an instrument of the free expression of desires. Young people have free reign of their bodies, allowing them to experience sexual pleasure with a spontaneity unencumbered by cultural and reproductive restrictions (Giddens, 2004).

At the same time, as pleasure is pursued, there is a relaxation of the norms of sexual exclusiveness between partners sexual exclusiveness here has a role in the relationship to the degree to which the partners mutually deem it desirable or essential (Giddens, 2004, p. 64). Although promiscuity is negatively assessed as a source of problems in young people, our study also reveals a certain degree of tolerance toward casual relations, or toward sexual relations with third parties even in the context of a stable relationship, especially for those under the age of 25. Reproduction is no longer linked to sexuality and relationships, although this aspect was more relevant in Spain and Portugal than in other countries.

Another noteworthy aspect of the results is the relevance of masturbation: young people report it as a habitual practice, although it appears undervalued. This perspective is perhaps still conditioned by orthodox religious positions and traditional cultural constraints that associate it with multiple negative connotations (Patton, 1986). Due to these ideas, circulated in Western society for decades, in many situations (for example, a stable relationship), masturbation generates feelings of sexual guilt (Santos et al., 2013).

In conclusion, our findings point to the need to expand young people’s knowledge and awareness of the complexity of sexuality and sexual health, linking it to emotional and relational aspects. It is necessary to implement CSE, as recommended in the guidelines of the World Health Organization [WHO] (2018) and United Nations Educational Scientific and Cultural Organization [UNESCO] (2018), that corrects stereotypes about diversity or equality that persist in educational institutions (Bejarano, 2017). The goal is to provide young people with knowledge, skills, attitudes, and values that empower them, allowing them to enjoy sexuality in a safe, healthy way and with a high level of long-term emotional well-being. Our study shows that although the sexual practices of young university students are more open and diverse today, they are not free from conflicts and problems associated with the experiences they entail. There is a clear dissociation between the reported behaviors and the values with which these behaviors are judged or perceived. The social and cultural context defines the mental framework from which one’s own sexual relations are experienced according to hegemonic social normativity. This is reflected in the fact that issues such as sexual diversity, equality in relationships between men and women, and practices outside the predominant heterosexual models did not have a very central position in any of the dimensions analyzed. On the other hand, the study also indicates a divergence among expectations regarding emotional relationships, love, reproduction, and sexual pleasure.

A comprehensive, complex and pluralistic vision of sexuality, as reflected in the baseline categories of the WHO and UNESCO in this study, would mean the integration of all these aspects into sexual experiences. This would result in a higher level of emotional well-being in young people by encouraging them to explore sexuality in a way less conditioned by the sociocultural parameters still in force. According to the World Health Organization [WHO] (2010), the resistance to CSE starting in childhood stems from a prejudiced assessment thereof; as a result, sexuality is still approached tangentially in academic and social education in most societies. As demonstrated, sexuality education does not imply greater sexualization at an early age, but quite the opposite: it ensures a higher level of health and safety, better preparation to position one’s sexual experience in a respectful relational framework, and acquisition of solid knowledge that allows one’s own identity and sexual experience to be explored autonomously during this stage of life.

## Data Availability Statement

The datasets generated for this study are available on request to the corresponding author.

## Ethics Statement

Ethical review and approval was not required for the study on human participants in accordance with the local legislation and institutional requirements. Written, informed consent was inferred through the completion of the questionnaire.

## Author Contributions

All the authors of this article have made an equal contribution to its completion and to the research process: literature review, methodological and instrument design, field work, analysis, and discussion of the results.

## Conflict of Interest

The authors declare that the research was conducted in the absence of any commercial or financial relationships that could be construed as a potential conflict of interest.
